# A Recurrent Temporal Model for Semantic Levels Categorization Based on Human Visual System

**DOI:** 10.1155/2021/8895579

**Published:** 2021-04-29

**Authors:** Mohammad Hossein Karimi, Reza Ebrahimpour, Nasour Bagheri

**Affiliations:** ^1^Faculty of Electrical Engineering, Shahid Rajaee Teacher Training University, P.O. Box: 16785-163, Tehran, Iran; ^2^Faculty of Computer Engineering, Shahid Rajaee Teacher Training University, P.O. Box: 16785-163, Tehran, Iran; ^3^School of Cognitive Sciences (SCS), Institute for Research in Fundamental Sciences (IPM), Niavaran, P.O.Box. 19395-5746, Tehran, Iran; ^4^School of Computer Science (SCS), Institute for Research in Fundamental Sciences (IPM), Farmanieh Campus, P.O. Box: 19538-33511, Tehran, Iran

## Abstract

Humans can categorize an object in different semantic levels. For example, a dog can be categorized as an animal (superordinate), a terrestrial animal (basic), or a dog (subordinate). Recent studies have shown that the duration of stimulus presentation can affect the mechanism of categorization in the brain. Rapid stimulus presentation will not allow top-down influences to be applied on the visual cortex, whereas in the nonrapid, top-down influences can be established and the final result will be different. In this paper, a spiking recurrent temporal model based on the human visual system for semantic levels of categorization is introduced. We showed that the categorization problem for up-right and inverted images can be solved without taking advantage of feedback, but for the occlusion and deletion problems, top-down feedback is necessary. The proposed computational model has three feedback paths that express the effects of expectation and the perceptual task, and it is described by the type of problem that the model seeks to solve and the level of categorization. Depending on the semantic level of the asked question, the model changes its neuronal structure and connections. Another application of recursive paths is solving the expectation effect problem, that is, compensating the reduce in firing rate by the top-down influences due to the available features in the object. In addition, in this paper, a psychophysical experiment is performed and top-down influences are investigated through this experiment. In this experiment, by top-down influences, the speed and accuracy of the categorization of the subjects increased for all three categorization levels. In both the presence and absence of top-down influences, the remarkable point is the superordinate advantage.

## 1. Introduction

The object categorization is a quick and accurate process in humanity's daily life which is surprisingly happening very effortlessly. The humanities' power in solving the problem of object categorization has challenged even the most advanced object recognition algorithms [1]. Therefore, human superiority over pattern recognition algorithms made the perception of neuronal system mechanism an exciting research topic over decades. To study the different visual cortex layers and understand its hierarchy structure, the processing time of the visual cortex is a useful tool. Note that the processing time in different parts of the visual cortex is dependent on the types of problems. Also, image processing is carried on in the visual cortex quickly and can be divided into two parts, namely, the primary processing and the high-level processing [2].

The visual system has a hierarchical structure in the brain that has a bottom to top hierarchical connections. This construction begins from the area called V1, which get the information from the LGN. Next, the visual system function is divided into two paths, called dorsal and ventral, respectively. In the following of the mentioned hierarchical process, V2, V4, and IT are the areas located in the ventral pathway, and in the dorsal pathway, V3, V5, and MT areas exist. Next to the mentioned structure, there are feedback links in all visual areas, even there are recurrent processing from V1 to LGN and retina that cause the top areas affection on the bottom areas which resulted the recurrent processing. Neurons are continually being adjusting in the human visual system [3–5].

According to the perceptual work involved in different tasks, the neurons adjusting process which is done by the mentioned areas in the visual system using the top-down influence may change, even when the stimulus remains unchanged [6].

Depending on the type of task, the alternations in the neurons adjusting are also seen in other brain areas like auditory [7]. The neurons adjusting in the lower area is affected by the top-down influences so that neurons provide reliable information for higher levels to identify the challenging stimulus. It should be noted, during stimulus detection, the neurons firing rate will increase by the feedback, depending on the characteristics of the stimulus. By counting this firing rate using recorder neurons, the visual system detects the stimulus [8].

The existence of top-down influences in the visual system causes different behavioral effects like attention, perceptual task, and expectation [9, 10]. All these effects help to create a consistent image of the stimulus in a sophisticated context in the visual system. In the case of attention, the participants are looking for a particular object in the field of view which is happening in the bottom-up and top-down influences. In expectation, there are no target features in the field of view, and after a time-lapse and top-down influence creating, the visual system can detect the target features with high accuracy. In the perceptual task, when the participants decide to identify a specific object, the visual system readjusts its characteristics for that task [11].

The semantic levels are divided into three levels of the superordinate (e.g., vehicle), the basic (e.g., ground vehicle), and the subordinate (e.g., car) [12]. The most practical semantic level is the basic level in which the humanities' daily life categorizations are mainly covered by this level. The basic is an intermediate level and requires a moderate expertise degree for the classification process. Meanwhile, the superordinate level is the highest level of categorization and requires very general information for the categorization. For example, by observing a wheel, an object like a vehicle can be imagined, but the type of vehicle cannot be recognized and it would require more features for type disclosure. Lastly, the hardest semantic level categorization is done at the subordinate level, which is the lowest level of categorization and requires very detailed information about the object for the categorization [13].

In 2008, the categorization paths for the classification of semantic levels in the visual system are studied by Mack and Gauthier [14]. In this study, the stimuli were shown to the participants in the up-right and inverted manner, and they compared the superordinate and the basic level in their study. The result of the experiments showed that two levels are classified in separate paths of the visual cortex.

In 2014, several experiments were conducted by Poncet and Thorpe to examine different classification of semantic levels [15]. In these experiments, the variety of stimuli and the interstimulus interval (ISI) have been studied, in which long and short ISI indicates the presence of feedback paths and the absence of feedback paths, respectively. In all of these experiments, the superordinate advantages were expressed.

In 2015, an experiment was organized by Mack and Palmeri, where the stimulus exposure time ranged from 25 to 250 milliseconds [16]. Their results showed that the superordinate and the basic advantages occurred at short and long exposure time, respectively. They reasoned that the time course of perceptual encoding would probably not be related to the speed of categorization decisions.

In 2016, in a report, the basic and the superordinate levels were reviewed by Vanmarcke et al. [17]. They changed the ISI from 20 to 80 milliseconds and asserted that, after 60 milliseconds, the top-down influences have been created and also the superordinate advantages have been observed during experiments. In this research, for long stimulus display time, the feedback paths which rearrange neurons in lower areas are established.

In 2019, some experiments on models and humans were performed by Xu et al. [18]. They express that the efficiency of categorization depends on some factors such as top-down influences, levels of abstraction, and target image size relevant (aspect ratio) to the entire image. They also mentioned that there is a challenge between the stimulus presentation time and the accuracy in such a way that by increasing the stimulus display time, the efficiency goes up and vice versa. During these experiments, the results were saliently different about the face because of the unusual location of facial processing in the brain and also the differences in feedback paths.

In 2019, Rajaei et al. studied the occlusion issue and its effect on the entry of feedback paths into the categorization process [19]. They checked the decoding accuracy by MEG signals and examined three levels of occlusion as zero, 60, and 80 percent. Their results indicated that more top-down influences are entering the categorization with more increase in occlusion.

A review of the levels of categorization in case of the accuracy and the classification speed can assist to understand the functionality of the human visual system. It can potentially explain the classification mechanisms in the visual cortex and address the questions like which level of classification is faster and which level is more accurate, and it can describe the mechanism of the top-down influence. Of course, it should be noted that cases in this field have been studied in great detail. One of these is the effect of the upper surface structure on the lower surface category [20]. They examined animacy against variability in the classification of animals and instruments, and they concluded that the speed of information processing is determined by the intraitem variability and is not related to the animacy. Also, in the future, the intraitem variability and frequency effect of the name can be examined simultaneously with the effects of feedback and category level.

In 2013, Ilic et al. investigated the effect of the superordinate category structure on the subordinate label verification in the absence of animation.

The rest of this paper is organized as follows. First, in section 2, we demonstrated a psychophysical experiment performed on ten participants. Here, we are asking the participants to categorize the objects like vehicles, ground vehicles, and cars in accordance with a label observed through each process in short and long ISI. Note that vehicle, ground vehicles, and car categories are, respectively, considered as the superordinate, basic, and the subordinate levels. These categories are selected as whole to fine, and other similar items can be considered. The experiment results illustrated the superordinate advantages in both short and long ISI. Then, in section 3, we propose a temporal model based on the human visual system that has feedback and the capability to simulate the top-down influences, which is training for ten object categories. Then, for the evaluation and validation of the proposed model, four experiments are performed, i.e., the up-right, the inverted, the occlusion, and the deletion images. In the model results, the superordinate advantages are seen and the top-down influences in the accuracy of up-right and inverted images are observed fewer while in occlusion and deletion images the accuracy was high. A possible reason for obtaining these results in the model can be that more neurons are activated in the categorization of higher levels. Finally, in section 4, conclusion and discussion of the paper are presented.

## 2. Materials and Methods

### 2.1. Participants

In total, ten volunteer participants, including seven males and three females, were participated in this experiment. The age of participants ranged from 20–31 years old, and the mean age was 23 years old (one left-handed). All participants had normal or corrected to normal vision and provided written informed consent. Also, all participants in this study speak Persian (mother tongue). They used common names for these objects. Initially, all participants were fully trained by images other than the main test images. The experiment was approved by the ethics committee of the Iran University of Medical Sciences.

### 2.2. Dataset

**Figure 1:**
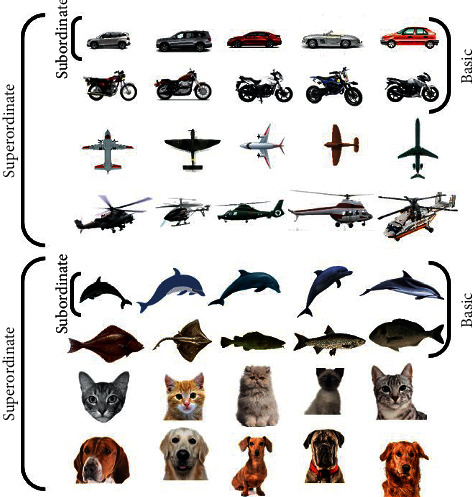
Some stimuli were used in all experiments (by row: cars, motorcycles, airplanes, helicopters, dolphins, fishes, cats, and dogs). The images were grayscale in an isolated condition. Each row is in the subordinate level, and each two row's burst indicates the basic level, and every four rows in the tandem shows the superordinate level.

We used gray images (255 × 255 pixels and subtended approximately about 7°×7° of visual angle) of eight object categories, including fish, dolphins, cats, dogs, airplanes, helicopters, motorcycles, and cars. Most of the images were chosen from the Chandler dataset [21], and other images were gathered from some available public sources on the Internet. The categorization levels were considered as vehicle/nonvehicle for the superordinate level, ground vehicle/nonground vehicle for the basic level, and car/noncar for the subordinate level.  Figure 1 illustrates several sample images, obtained from the dataset, for each individual task. The distractors consist of everything except the target. The subjects had no prior knowledge concerning the target in the picture.

**Figure 2:**
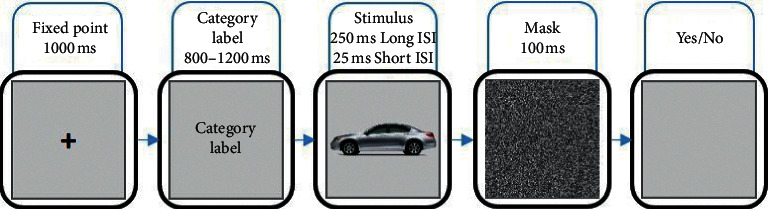
Psychophysics categorization task. A fixation point is illustrated for 1000 ms to focus on the subject. After that the categorization label is onset for 800–1200 ms. Next, a gray 256 × 256 image named stimulus is flashed for 250 ms for long ISI and 25 ms for short ISI, respectively, followed by a noise mask for 100 ms. At the end of the experiment, the subjects are asked to respond whether the presented image matched with the shown label or not, using “Yes” or “No” keys on a keyboard.

Each experiment started with a 1000 ms fixation point was used to centralize the participant's centralization followed immediately by vehicle, ground vehicle, or car category label image for 800 to 1200 ms. After that the test images were displayed 250 ms for long ISI and 25 ms for short ISI, respectively. Next, a noise image remained on the screen for 100 ms, followed by a blank until the participant responded (see Figure 2). Participants respond by pushing a “Yes” key if the label matches the object shown in the stimulus image and a “No” key if it does not. One-third of the category verifications were made at the superordinate level (car, motorcycle, airplane, and helicopter), one-third were made at the basic level (ground vehicles), and the other one-third were made at the subordinate level (car). Note that in a correct response, the category label and the object in the stimulus must be matched.

The experiment was conducted in a dark room, and the participants were placed at a distance of about 50 cm from the computer screen (Intel core 2 duo processor 2.66 GHz, 4 GB RAM, 85 Hz monitor refresh rate). Also, MATLAB software and psychophysics toolbox [22–24] were used to run this experiment. All images were divided into two blocks which contained 146 images (73 animals and 73 vehicles). Each participant responded to two different blocks of images (10 subjects × 2 blocks = 20 responded per blocks), and we obtained ten different reaction times per image.

**Figure 3:**
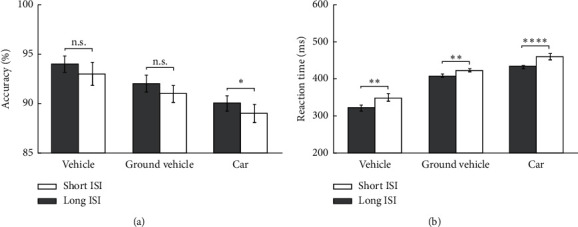
The accuracy and reaction time for the three levels of superordinate, basic, and the subordinate for short and long ISI. (a) The accuracy for three levels of categorization. It can be seen that the superordinate is more accurate than other levels for both long and short ISI. (b) Unlike accuracy, reaction times for superordinate level are less than other levels, which means the superordinate is the fastest level. These two images show the superordinate advantage for presence and absence of top-down influences.

The experiment results for all three categorization tasks show the high accuracy in the different levels, i.e., median 93% in the superordinate, 90% in the basic, and 89% in the subordinate for long ISI (see Figure 3(b)). As mentioned previously, we obtain ten different reaction times (RT) for each individual image. In the experiments, the correct responses with very high (>1000 ms) or very low (<200 ms) RT were considered as an outlier. Therefore, RTs higher than 1000 ms were eliminated from analyzation first. In addition, only images with more than six correct responses were analyzed. Here, RT for each individual image was computed as the median of RT among all correct responses.  Figure 3(a) shows the median, within-class, and between-class variance of RT. As it is obviously shown, the levels are separated enough regarding the average and variance of each level. The procedures at the superordinate level are faster than those at the basic level, and the procedures at the basic level are faster than those at the subordinate level. In this experiment, we found a superordinate level advantage in terms of categorization.

## 3. The Model

### 3.1. The Base Model

The spiking HMAX model presented by Masquelier and Thorpe in 2007 is one of the feedforward models that is used for objects classifying with high performance [25, 26]. In this model, the middle layer neurons selection is based on the characteristics of the frequent patterns of input data. These neurons become more reliable with increasing iteration in the learning phase, and more specifically, each neuron expresses a specific feature of the input data. This hierarchical model consists of four layers S1, C1, S2, and C2, which are described in detail in the following subsections.

#### 3.1.1. S1-Layer

**Figure 4:**
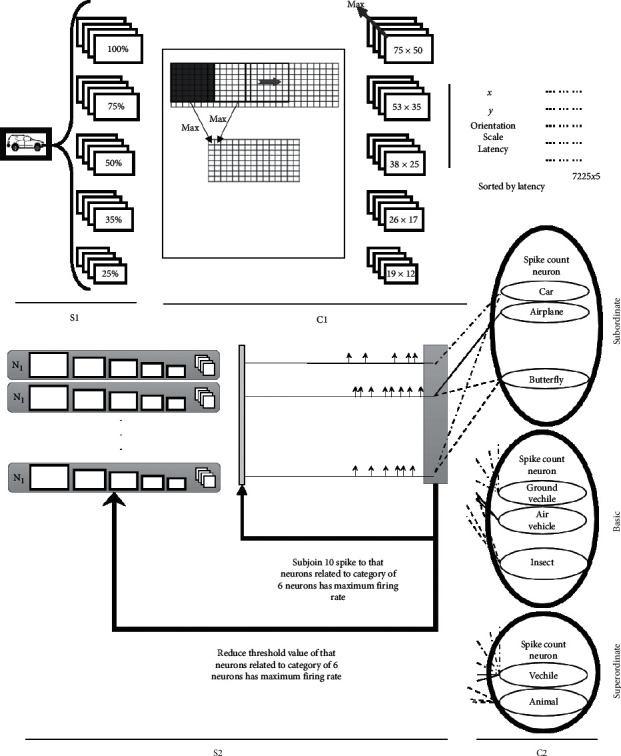
The proposed computational model. In this model, four layers are represented. The S1-layer refers to simple neurons in the v1 region and the edge detection process. The C1-layer points to complex cells in the human visual system; the remarkable points of this layer are the maximization as its main operator and the time appearance in it. The output of the C1-layer is an array of spikes, in which the time is based on the salient of the edges. The S2-layer cells are related to the intermediate visual system features. In this study, the S2-layer includes 110 cells, for ten categorizes. The layer performance evaluation is slightly different at the test and training phase in which the two feedbacks, related to expectation influence, occur on this layer. The C2-layer of the model is performing categorization at different levels and does this by counting the spikes.

The S1-layer represents the simple V1 vision cells in the human visual system, described by Hubel and Wiesel [27]. The S1 responsibility is to recognize the image edges in four directions and five different scales. In this layer, the image edge-recognition process is done by a convolutional operator in four directions with the angles of 0 + 22.5, 45 + 22.5, 90 + 22.5, and 135 + 22.5 in degree radius (to prevent focusing on the horizontal and vertical edges, the 22.5-degree rotation is considered). In addition, these filters are applied to the input image in 5 scales, i.e., 100%, 75%, 50%, 35%, and 25%, respectively. Hence, as it is shown in Figure 4, the S1 output includes 20 images.

#### 3.1.2. C1-Layer

On this layer, the output images of the S1-layer are covered by a 7 × 7 frame, which move on to them with a unit overlapping, and every time after frame deployment, the maximum ratio in this frame is considered as a pixel of the output image; therefore, the output image per scale size at this stage is 1/6 of the size of the input image. Next, the maximum ratio between four orientations of all pixels has picked up for all five mentioned scales. So, a matrix is considered to store all pixels with their scales, directions, coordinates, and latency, in which the inverted value of that maximum pixel is equal by the pixel's latency. Finally, a sorted 7225 × 5 matrix which includes scale, orientation, coordinate, and latency is obtained and sorted based on the latency. Following these stages, the time will appear in the model, which is dependent on the edge salience of the input image and causes a new definition as C1-layer's spike which is the label of the all five horizontal elements of a 7225 × 5 matrix.

#### 3.1.3. S2-Layer

The S2-layer cells are representing the central features of the visual system. In this study, the number of neurons is considered to be 110 for this layer, in which the neurons count is based on the best efficiency for ten categories. Each neuron contained five matrixes with the sizes 75 × 50, 53 × 35, 38 × 25, 26 × 17, and 19 × 12, respectively, and also four weight matrixes with the size of 16 × 16. In the training phase, the element's amount of weight matrixes has a randomized initial value between 0 and 1. By each spike-entrance from the previous stage, according to the spike direction, one of the four weight matrixes is selected to be added with one of the five neuron matrixes according to the scale and the coordination of the previous spike. In a way of spikes consecutive arrival, the magnitudes of the matrices in the neurons located in S2-layer increase. When the amount of a matrix element reaches the threshold value (based on the maximum efficiency, the threshold value considered as 64), a fire occurs. At this moment, according to the STDP rule, the weight matrix that caused the fire is updated. In the testing phase, everything is similar to the training phase, with the difference that in the firing moment the weight matrix is not updated anymore, but, instead, its time is store in a matrix called the spike series matrix.

In this layer, also there are two feedbacks that are responsible to model the expectation influence at the testing phase. After the spike series creation in the layer's output, the model chooses the number of 6 neurons with the highest firing rate value. The firing rate of the neurons, which are dependent on the mentioned neurons category, decreases as much as four-units and in simultaneous action, and 10 spikes will be added to the spike series matrix in every feedback loop running (the feedback loop is run for eight times).

**Figure 5:**
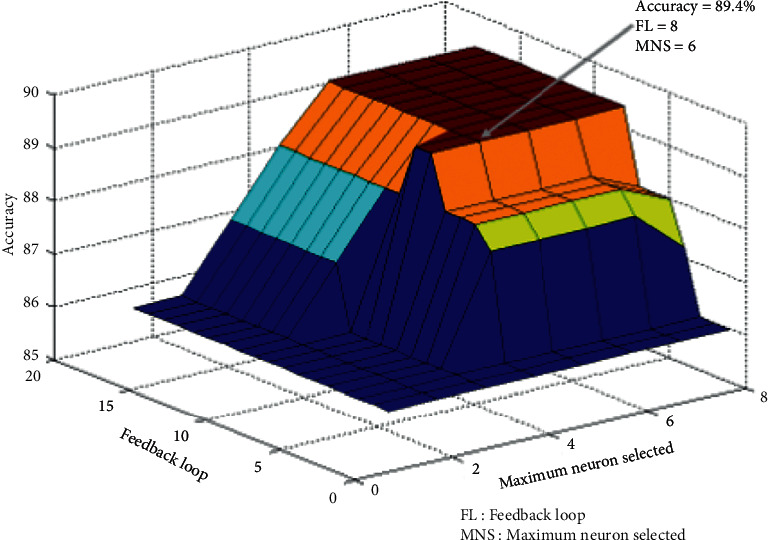
Choose the best number of feedback feeds and the best number of feature neurons participating in the feedback. To select the number of feedback loops and the number of feature neurons, the model has executed ranges from 1 to 20 feedback loops for the number of feature neurons between 1 to 8 neurons. There was no difference in accuracy increment after running the number of 8 feedback loops and the number of 6 feature neurons, and only the computational load of the model increased.

All the values, e.g., the number of selected maximum neurons, the feedback times, the value of firing threshold reduction, and the added spike numbers per feedback loop are selected based on the maximum efficiency at the subordinate level.  Figure 5 shows the median accuracy of the model based on the feedback loops and the maximum selected neurons.

#### 3.1.4. C2-Layer

The classification method ends in the C2-layer, and by looking into its structure, three feedback paths can be found that will be discussed in the following. The influence of perceptual task is covered by one of these feedbacks, and the influence of expectation is covered by two other ones. The decision about the categorization level is the first feedback responsibility, and along with changing in categorization level, the layer structure will change either.

**Figure 6:**
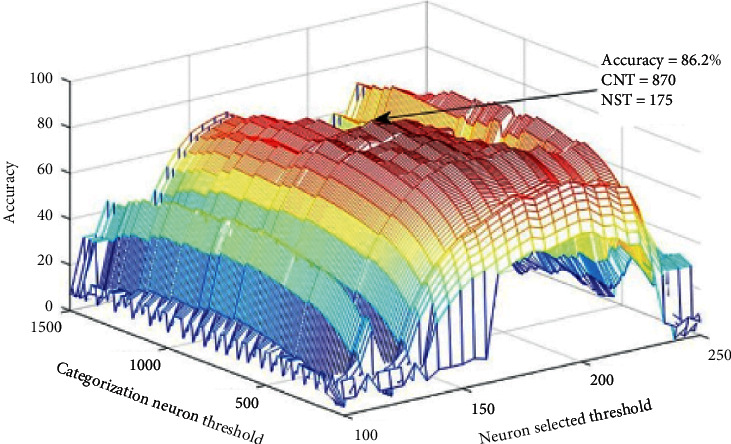
The threshold amount (NST) changed between 100 and 250 with intervals of 5 among them to find the best threshold for selecting best neurons in each category, and the threshold amount (CNT) changed between 10 and 1500 with intervals of 10 among them to find the best threshold for spike count neurons. In each step, the median accuracy is calculated for all categories, and the best thresholds (NST) for choosing neurons were 175 and for neurons counting (CNT) were 870.

To perform the classification process at the subordinate level, the neurons dependent on each category's feature in the previous layer are connecting to a spike count neuron. At the basic level, the neurons that depend on two categories (e.g., car and motorcycle in ground-vehicle category) are connected to a spike count neuron according to its characteristics in other layers. Also, in the superordinate level, the neurons associated with more than two categories are connected to the spike count neuron. In the S2-layer, a general threshold has been defined for each neuron's firing rate, to select the neurons associated with each category. In the C2-layer, there is a threshold level for spike count neurons to classify the categories.  Figure 6 shows these two threshold values to obtain the maximum amount of accuracy at the subordinate level without feedback.

In the S2 and C2-layer, respectively, to determine the neurons that are associated with each category and to classify the categories, two general thresholds have been defined in the present paper which are denoted by NST (Neuron Selected Threshold) and CNT (Categorization Neuron Threshold), respectively, and the results of these two thresholds are shown follows.  Figure 6 shows the two mentioned threshold values, to obtain the maximum amount of accuracy at the subordinate level without feedback.

In Figure 4, the proposed model is illustrated with three feedbacks, in which one of them stated in the C2-layer and the two other ones in the S2-layer.

**Figure 7:**
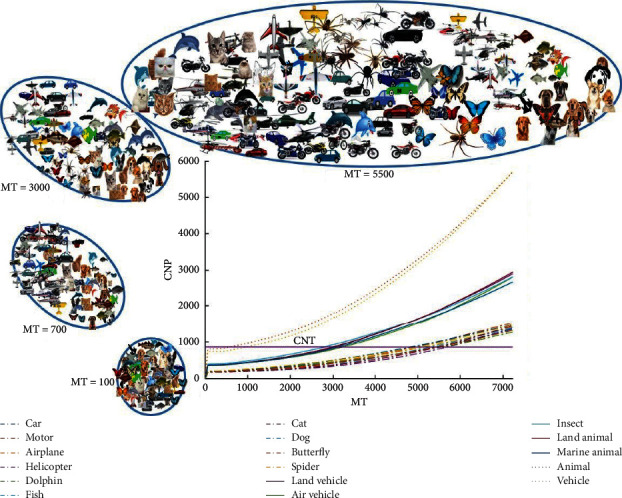
The potential of neurons categorization, for different objects at three levels of classification, is shown over time. These categories are separated from each other in a way that the classifier would be able to recognize them alongside of the time-increasing. There are 10, 5, and, 2 categories in the subordinate, basic, and superordinate levels, respectively. The shapes around the graph depict the separation of the categories at times 100, 700, 3000, and 5500 using a k-means classifier, and the notable point is that the assumed time is the model time, which ranges from 0 to 7225.

By the entrance of each spike from previous related neurons, the potential of the neurons categorization in the last layer is increased by one unit. When this potential reaches the threshold value (CNT = 870), the neuron fires and the input image is allocated to the fired neuron's category.  Figure 7 shows the neurons category potentials in the feedback mode. By increasing the time and arrival of the spikes from the related neuron's category, the potential of the neurons categorization rises, and the classification of categories can be observed over time.

**Figure 8:**
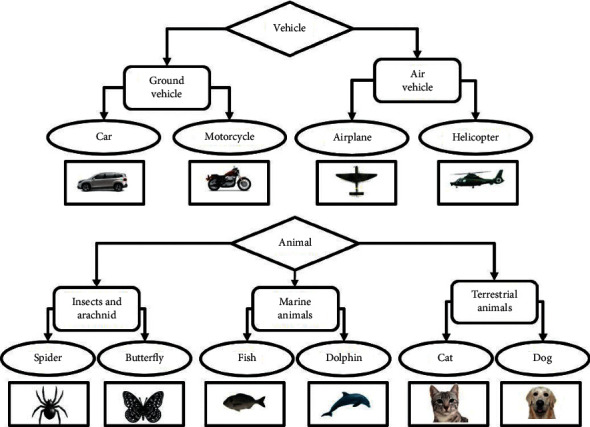
Ten categories of images are used to train and test the model. The images of cars, motorcycles, helicopters, fishes, and dolphin images are all inside view; the images of cats and dogs are in front view; and airplanes, spiders, and butterflies are in top view. There are also, respectively, 10, 5, and 2 categories at the subordinate, basic, and superordinate levels in the dataset.

In the training phase, ten categories of objects are used, for selecting neurons that are related to each category, and in the model's testing phase, 200 images are considered for each category. The categorization process should be done next, aiming to have three levels of classification. The illustration of three categorization levels is shown in Figure 8. The figure shows that 10 categories are considered for subordinate level, i.e., cars, motorcycles, airplanes, helicopters, cats, dogs, dolphins, and fishes, and ground vehicles. On the other hand, the basic level includes air vehicles, terrestrial animals, marine animals, and insects. Finally and in a more general view, vehicles and animals are considered as superordinate level.

In this section, the number of four experiments has been designed for the test and the model evaluation, in which the first experiment is about the up-right image series, the second one is for the evaluation purpose of inverted images, and the third and fourth experiments are about the occlusion and deletion images, respectively. In the two last experiments, because of the experiment's hardness, the top-down influences have more effective roles. In the following subsections, the experiments are described in detail.

### 3.2. Experiment 1: Up-Right Objects

In the first experiment on up-right objects, to test the proposed model, images were applied to the model without any change. It is expected to reach better results compared with the feedback-less mode because some neurons have not reached the threshold value *i* in that mode, and their firing rates are higher now in the comparison with feedback-less mode. So, they reach the threshold value with feedback's help, and this is interesting to say that the correct features reported by these neurons.

**Figure 9:**
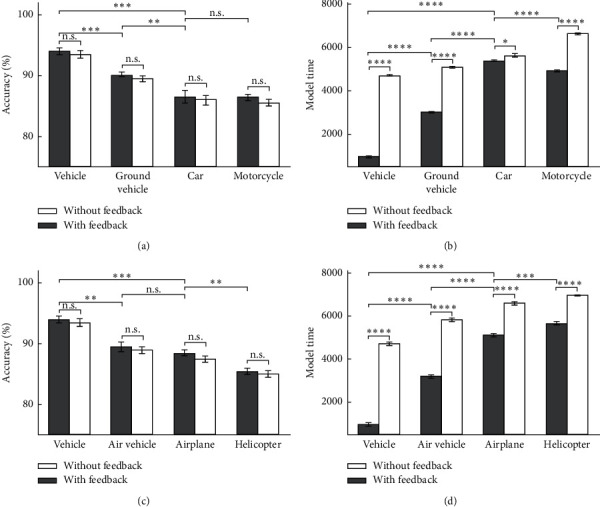
The model's accuracy and the model's time for vehicle images as input in 10 runs of the feedback and feedback-less mode for up-right images in three levels of categorization. (a) The model's accuracy of car and motorcycle at up-right images. (b) The model's time of car and motorcycle's up-right images. (c) The model's accuracy of airplane and helicopter's up-right images.(d) The model's time of airplane and helicopter's up-right images for the feedback mode and feedback-less mode.

**Figure 10:**
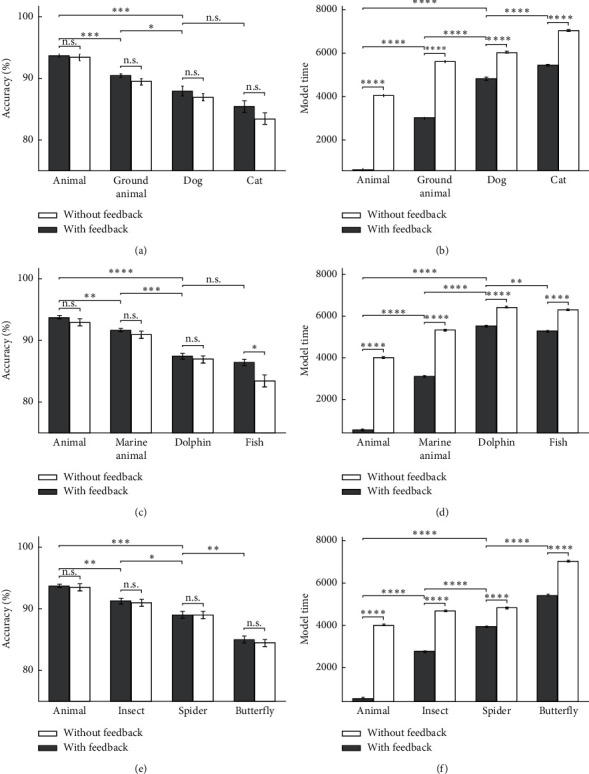
The model's accuracy and the model's time for animal images as input in 10 runs of the feedback and feedback-less mode for up-right images in three levels of categorization. (a) The model's accuracy of dog and cat's up-right images. (b) The model's time of dog and cat's up-right images. (c) The model's accuracy of dolphin and the up-right images of fish categories. (d) The model's time of dolphin and fish's up-right images. (e) The model's accuracy of spider and butterfly's up-right images. (f) The model's time of spider and butterfly's up-right images.

Figures 9 and 10 show the model's accuracy and the model's time for two modes of feedback-less and with feedback in up-right images. As it is obviously shown, the accuracy of the proposed model increases in most categories and levels, and the time is reduced. It can be seen that whenever the accuracy value is less in the feedback-less mode, more feedback effect is reachable. It should be noted that the feedback effects on accuracy in up-right images were not very significant, exclude for the fish categorization which was significant as an expectation. The model's accuracy and the model's time for the vehicle category in the up-right images are illustrated in Figure 9. In Figures 9(b) and 9(d), in a high scale, the rising firing rate in the feedback mode reduces the model time to recognize a vehicle category because the spike count neurons are reaching the threshold value very quickly. Due to the different neurons accumulation in different categories, the categorization speed at higher levels is much faster than that at lower levels. In Figure 10, the model's accuracy and the model's time are shown for the animal categories in the up-right images. As it is obvious, the only category for which the accuracy has a significant change in feedback mode, compared with feedback-less mode, is the fish categories. The reason could be its low accuracy in feedback-less mode.

### 3.3. Experiment 2: Inverted Objects

**Figure 11:**
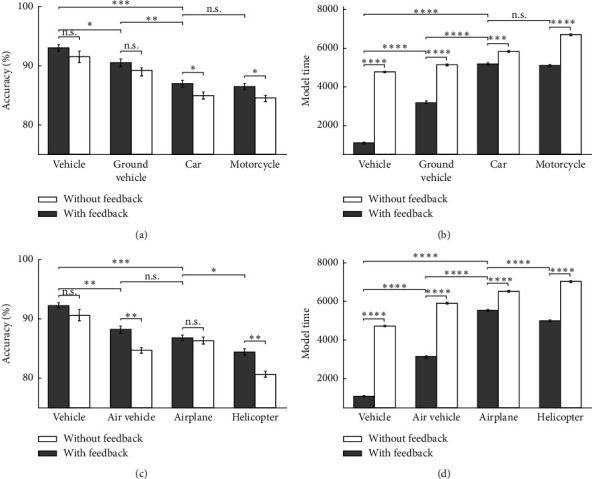
The model's accuracy and the model's time for vehicle images as input in 10 runs of the feedback mode and feedback-less mode for inverted images in three levels of categorization. (a) The model's accuracy of car and motorcycle's inverted images. (b) The model's time of car and motorcycle's inverted images. (c) The model's accuracy of airplane and helicopter's inverted images. (d) The model's time of airplane and helicopter's inverted images.

In this experiment, the inverted objects are chosen as the model's input in a way that images are inversely applied to the model. This experiment aims to test and evaluate the correctness, feedback effects, and the categorization levels arrangement of the model for object recognition in the case of having untrained inputs. As a result, the inverted images have shown the impact of feedback more than up-right images in different categories at different levels. The model's accuracy and the model's time for vehicle categories are illustrated in Figure 11. By further analyzing, we observed that, for all categories excluding the airplane category, there are significant differences in the accuracy between feedback-less and feedback modes. The reason for accuracy-rising is that the feature's firing rates that describe the categories are very low for inverted images. In the case of model time analyzing, due to the very high firing rate in the feedback mode, the model times are extensively decreased. Also, in the inverted image modes, the categorization levels arrangement can be seen as the superordinate, basic, and the subordinate levels, respectively.

**Figure 12:**
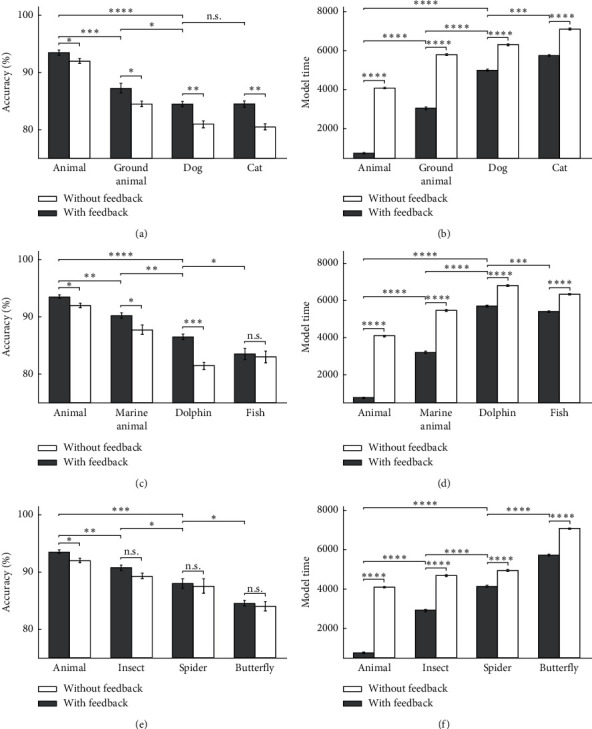
The model's accuracy and the model's time for animal images as input in 10 runs of the feedback mode and feedback-less mode for inverted images in three levels of categorization. (a) The model's accuracy of dog and cat's inverted images. (b) The model's time of dog and cat's inverted images. (c) The model's accuracy of dolphin and fish's inverted images. (d) The model's time of dolphin and fish's inverted images. (e) The model's accuracy of spider and butterfly's inverted images. (f) The model's time of spider and butterfly's inverted images.

**Figure 13:**
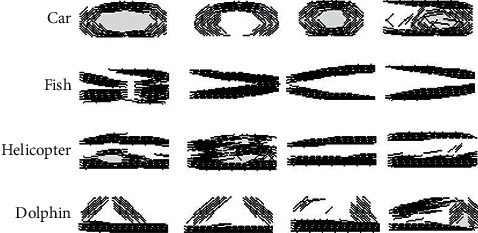
Some features related to the car, fish, helicopter, and dolphin categories are obviously shown in which these features represent the wheels for car categories and the dorsal, ventral, and caudal fins for fish categories. The image inverting does not affect these features. However, selecting features like the cabin's overall shape and the dorsal fin for helicopter and dolphin categories, respectively, indicate the situation where the input inverted image cannot be recognized.

In Figure 12, the model's accuracy and the model's time for animal categories are shown, in which the accuracy decreases sharply in cases that the category's related features fail to provide the correct answer for input images in inverted mode.  Figure 13 shows some of the describing feature neurons for some of the categories. For example, there is a sharp drop in the accuracy in the case of dolphin categories in feedback-less mode with inverted images because the related feature neurons are mostly dependent on the dorsal fin, which are lost by inverting the input image, while for fish categories, the result is different. In the case of the car and the motorcycle categories, the accuracy drop is also lower due to some important feature existence like wheels that the inversion in the shape of the wheels have no effect on image formation.

### 3.4. Experiment 3: Occluded Objects

**Figure 14:**
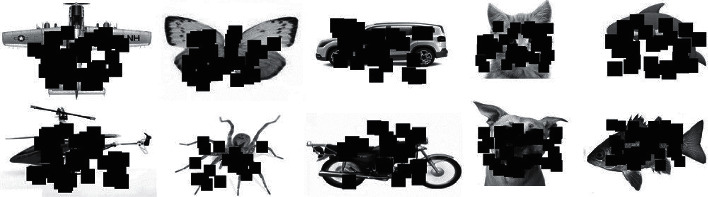
The occluded images for the ten categories. These images are shown with 40 to 60 occlusion percent, which are created randomly and used to test the effect of feedback in the case of occlusion.

The object recognition process with occluded images is more complicated, and a feedforward model will be able to solve this problem efficiently. In the proposed model, the neuronal firing rate which describes each category features is significantly reduced by the obstruction's impact. The reason is that these neurons' features, which are dependent on the characteristics of each category, will disappear by applying the occlusion mode on input images. The occlusion data are generated in a way in which some black spots are randomly placed on the images to calculate the occlusion percentage. The occlusion's percentage results from the division of object pixels that are covered by black spots and the total number of object pixels. In this experiment, the occlusion image percentages fluctuated between 40 to 60.  Figure 14 shows some of the occluded images for the ten categories.

With occlusion, the firing rate of the neurons, that describes the object's characteristic, reduces extensively such that the potential of spike count neurons does not reach the threshold, and classification disorder in many cases may happen. Hence, in the provided situation, the accuracy decreases sharply for occlusion mode. In the categorization of occluded images, the feedback paths will be very effective because these paths cause an increase in the firing rate of the features' neurons that are eliminated due to the occlusion effect. Hence, the spike count neurons reach the threshold value.

**Figure 15:**
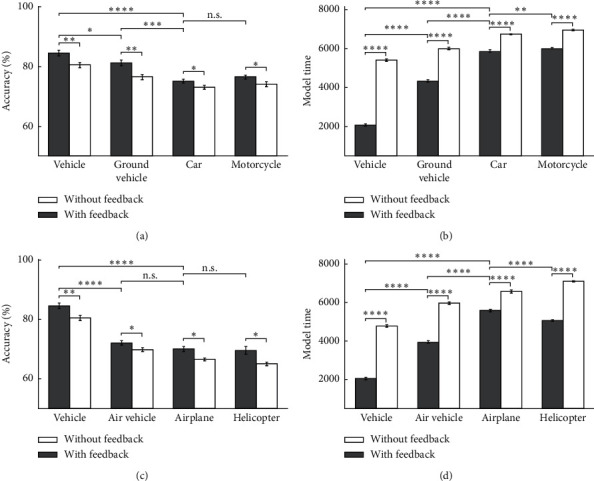
The model's accuracy and the model's time for vehicle images as input in 10 runs of the feedback mode and feedback-less mode for occluded images in three levels of categorization. (a) The model's accuracy of occluded images of car and motorcycle. (b) The model's time of occluded images of car and motorcycle. (c) The model's accuracy of occluded images of the airplane and the helicopter. (d) The model's time of occluded images of the airplane and the helicopter.

The model's accuracy and the model's time for occluded images, in the vehicle category, are illustrated in Figure 15. As it is obviously shown, the accuracy in the feedback-less mode was reduced significantly, and the maximum accuracy at subordinate levels was below 70 percent.

The highest accuracy dropping is related to the helicopter category, and the lowest one is in the motorcycle category. In the case of the helicopter, the reason for this reduction is that the neurons which are responsible to describe the helicopter characteristics are affiliated with larger and more general cases of the helicopter. For the motorcycle category, the reduction comes from the fact that the neurons which are describing the motorcycle characteristics are dependent on the smaller and more specific features.

In this experiment, the top-down influences cause accuracy-increasing in all three levels. In the proposed model, the reason for accuracy improvement is the increase in firing rate for those objects in which some of their features are occluded.

**Figure 16:**
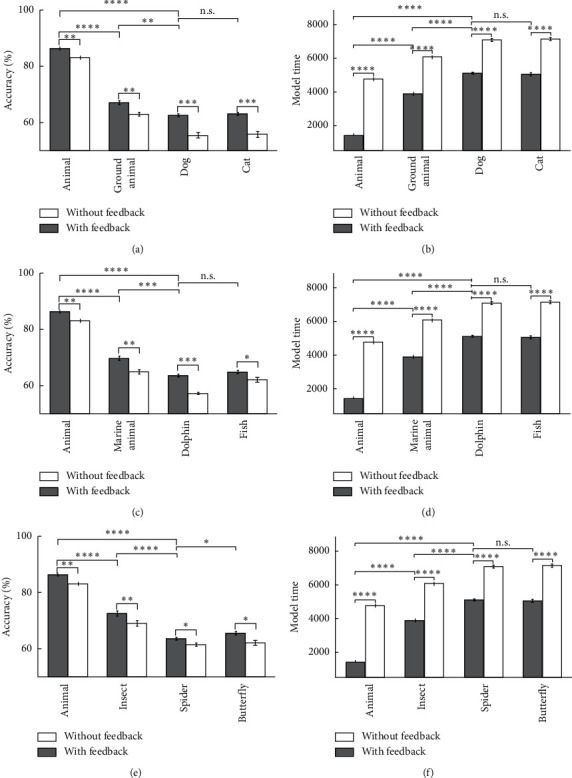
The model's accuracy and the model's time for occluded images of animals as the input in 10 runs of the feedback mode and feedback-less mode in three levels of categorization. (a) The model's accuracy of occluded images of dog and cat. (b) The model's time of occluded images of dog and cat. (c) The model's accuracy of occluded images of dolphin and fish. (d) The model's time of occluded images of dolphin and fish. (e) The model's accuracy of occluded images of spider and butterfly. (f) The model's time of occluded images of spider and butterfly.

The model's accuracy and the model's time for occluded images in the animal category are illustrated in Figure 16. For the animal categories, all the assumptions are similar to those of the vehicle categories. In animal case, also the accuracy and speed are falling, where lots of their mineral features were acquired by the model in the training phase, in comparison with the categories in which their general features that are trained by the model are fewer. Hence, the top-down influences can increase the accuracy and reduce the time of object detection at the model in any case.

### 3.5. Experiment 4: Deletion Object

**Figure 17:**
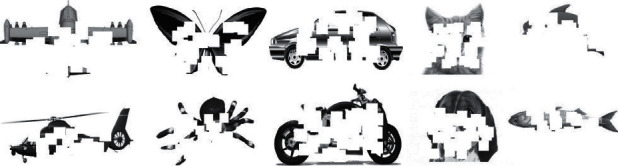
The deletion images for the ten categories. These images are shown with 40 to 60 deletion percent, which are created randomly and used to test the effect of feedback in deletion.

In this experiment, which is based on deletion images, some of the object's features are removed from the input images. This experiment's result has the most similarity to the occlusion image experiment. The reason comes from the fact that in both of them, some features are not presented to the model, which has obvious effects in the experiment results. As it is shown in Figure 17, some object parts in the image are randomly deleted to construct deletion images, in which the deletion percentage in any image is between 40 to 60.

In this experiment, the object recognition speed in the presence of top-down influence is rising significantly, alike occlusion mode. Also, in feedback-less mode, the results of the experiment's accuracy decreased significantly. It is because the neurons related to deleted features are not spiking any more, which leads to a situation for categorization neurons that they will not be able to reach the threshold value. However, with the mechanism of the top-down influence, the unfaded features help to undo the faded features in the top layers of the model. In fact, the model reconstructs deleted features due to the object's unfaded features and creates a suitable firing rate for faded features.

**Figure 18:**
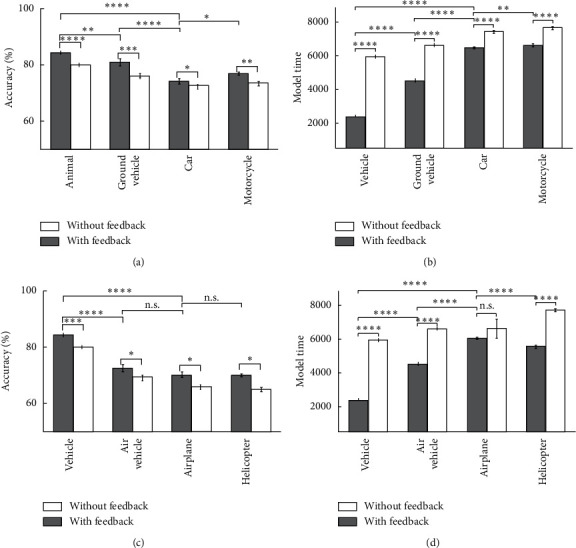
The model's accuracy and the model's time for deletion images of vehicle as the input in 10 runs of the feedback mode and feedback-less mode in three levels of categorization. (a) The model's accuracy of car and motorcycle's deletion images. (b) The model's time of car and motorcycle's deletion images. (c) The model's accuracy of airplane and helicopter's deletion images. (d) The model's time of airplane and helicopter's deletion images.

The model output results for the vehicle categories are shown in Figure 18. The accuracy and recognition's speed were significantly increased in all categories.

This increase in the accuracy at higher levels is due to the simplification of the classification problem and the more neurons involvement. Moreover, the accuracy-rising in the categories for that the model has been trained with mineral features which is more significant than the categories that are trained with more general features. For example, the accuracy-rising of the motorcycle category was higher that of than the helicopters.

**Figure 19:**
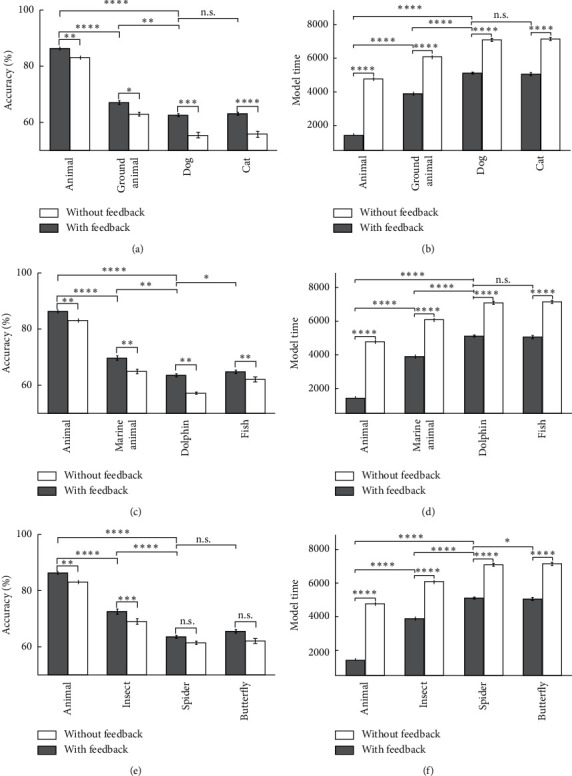
The model's accuracy and the model's time for animals as the input in 10 runs of the feedback mode and feedback-less mode for deletion images in three levels of categorization. (a) The model's accuracy of dog and cat's deletion images. (b) The model's time of dog and cat's deletion images. (c) The model's accuracy of dolphin and fish's deletion images. (d) The model's time of dolphin and fish's deletion images. (e) The model's accuracy of spider and butterfly's deletion images. (f) The model's time of spider and butterfly's deletion images.

At last, the deletion effects on the model's accuracy and the model's time with the feedback presence and absence in all level of categorization for animal categories were illustrated. As is obviously shown in Figure 19, there is a significant increase in the accuracy and speed in the presence of top-down influences in some categories, e.g., cats and dogs, that have had a severe reduction in accuracy and speed in deletion mode. Generally, the reason for significant accuracy-increasing in feedback mode for the animal categories is that the model has acquired these categories with more details.

## 4. Conclusion and Discussion

In this paper, the effects of top-down influences and level of categorization in the human visual system have been investigated. For this purpose, a task to study the human visual system on the categorization of objects has been designed, which examines the level of categorization and top-down influence. Due to this task which is designed to investigate the top-down influence of the human vision system, a long stimulus presentation time is considered to establish feedback paths at this time. The analysis of the experiment results has shown the lower detection time and higher efficiency in the categorization levels of the superordinate, basic, and the subordinate, respectively. In addition, a temporal model that has feedback and the capability to simulate the top-down influences was presented based on the human visual system, which can recognize objects with high accuracy and high speed. In the explained model, there are three types of feedback in which one of them represents the perceptual task and the expectation is expressed by the two other ones. To analyze the model performance validation, several experiments have been studied on different image-types, including up-right images, inverted images, occluded images, and deletion images. These experiments' results show that top-down influences have a significant impact on perceptual task and expectation issues. More precisely, to categorize an object in an up-right image or an inverted image, the feedforward paths could be sufficient and somewhat enough. However, for deletion and occlusion images, feedback paths are also required. Depending on the type of issue, feedback paths can be useful in the level of categorization, which means the vitality of top-down influences is less at higher levels. Still, in difficult topics such as analyzing the images including occlusion or deletion, even higher levels of categorization also require top-down influences to solve the problem.

According to Figure 10, in all categories except one of them, the establishment of top-down influences does not have a significant effect on the accuracy-increasing. The reason is that there is a little need for feedback path in an up-right image categorization, and a feedforward path is enough to solve the problem of categorization in this case. In up-right images, this occurs almost uniformly for all three levels, and only at the subordinate level, which is the most challenging classification level, little feedback paths are needed [28]. The classification task related to inverted image categorizations becomes a bit more complicated. The complication reason is that the neurons which recognize each class's feature have no information about the inverted image features, which of course have not been trained for reverse input images. For instance, the model has no perception of the dolphin's dorsal fin or overall body of the helicopter in inverted mode. Hence, the firing rate of these neurons decreases sharply in inverted mode; however, when the category includes rotation-invariant features, e.g., wheels in car and motorcycle, reversing the input images to the model did not affect the firing rate significantly and its identification performance did not decrease very much. The remarkable point is that the firing rate reduction in inverted mode is mostly compensated by top-down influence in feedback mode, and it creates a considerable increase in accuracy for some categories of inverted images. The classification's level in inverted images in the case of superordinate, basic, and subordinate is also alike to up-right images, in which this requirement to top-down influences is less evident [29, 30].

The last two experiments were dedicated to occlusion and deletion modes, respectively, in which top-down influences had the most impact as a result. In this article, these two experiments are the most difficult tests that we were able to show the feedback effect. In occlusion and deletion modes, establishing the top-down influences almost has compensated the accuracy rate reducing; it is also necessary to note that if some feature of an object is removed or hidden from the image, the neuron's firing rate associated with that feature will dramatically decrease. As a conclusion, the firing rate-drop also has an impact on the significant reduction in the potential level of the categorization neuron for occlusion and deletion objects, where the inability of the potential of categorization neuron to reach the expected threshold value results in a severe reduction in accuracy.

In the model with top-down paths, neurons firing rate of the objects with non-eliminated attributes helps to increase the firing rate of neurons with the eliminated attributes, which lead to reconstruct the features of occlusion or deletion. This also helps to increase the potential of the neurons clustering and model efficiency.

## Data Availability

The data used to support the findings of this study are obtained from the website of the Lab (http://ebrahimpourlab.ir/).
